# Delayed pregnancy disclosure, attributed social factors and implications for antenatal care initiation: a qualitative study among Ugandan women and their partners

**DOI:** 10.1186/s12978-025-02040-y

**Published:** 2025-05-23

**Authors:** Hadija Nalubwama, Alison M. El Ayadi, Cynthia C. Harper, Josaphat Byamugisha, Dilys Walker, Alexander C. Tsai, Blake Erhardt-Ohren, Umar Senoga, Paul J. Krezanoski, Carol S. Camlin, Alison B. Comfort

**Affiliations:** 1 Department of Obstetrics and Gynaecology, College of Health Sciences, School of Medicine, Makerere University, PO Box 7072, Kampala, Uganda; 2 Department of Obstetrics, Gynecology and Reproductive Sciences, Bixby Center for Global Reproductive Health, University of California San Francisco, 550 16 th Street, San Francisco, CA 94143 USA; 3 Center for Global Health and Mongan Institute, Massachusetts General Hospital, Harvard Medical School, 125 Nashua Street, Suite 722, Boston, MA 02114 USA; 4 University of California Berkeley, 2121 Berkeley Way, Berkeley, CA 94704 USA; 5 University of California San Francisco, Zuckerberg San Francisco General Hospital, 1001 Potrero Ave, San Francisco, CA 94110 USA; 6 Department of Obstetrics, Gynecology and Reproductive Sciences, Bixby Center for Global Reproductive Health, University of California San Francisco, 1330 Broadway, Suite 1100, Oakland, CA USA

**Keywords:** Pregnancy, Uganda, Antenatal care, Social support, Social networks

## Abstract

**Background:**

Pregnant women in Uganda access antenatal care later in pregnancy than recommended. One potential factor influencing timing of antenatal care initiation is delayed pregnancy disclosure affecting the social support needed to facilitate care-seeking. However, data exploring women’s decisions to disclose their pregnancy and the consequences for antenatal care-seeking are limited. We sought to understand social norms around pregnancy disclosure among pregnant Ugandan women and their male partners to inform interventions promoting appropriate antenatal care initiation.

**Methods:**

In August-October 2020, we conducted in-depth interviews with 30 pregnant women and 15 male partners attending their first antenatal care visit at Kawempe National Referral Hospital, Kampala, Uganda. The female participants were purposively selected for their varying partnership status. We asked study participants about social norms around pregnancy disclosure, to whom they disclosed their pregnancy and when, whether pregnancy disclosure influenced social support they received and its implications for antenatal care initiation. We analyzed transcripts using deductive and inductive thematic analysis.

**Results:**

Most participants selectively disclosed their pregnancy once confirmed, preferring to first disclose to close, trusted social relations (e.g. partner and/or mother), followed by a delayed selective, phased disclosure to other social relations (e.g., friends, certain family members and neighbors). Most women preferred waiting to disclose until at least their second trimester. Common reasons for delayed pregnancy disclosure included mitigating social judgement of pregnancy, fear of witchcraft and curses adversely impacting maternal and neonatal health, and concern about mockery or anger. These concerns arose from perceived social norms around the appropriate age for pregnancy and competing school priorities; lack of social recognition of the partnership and/or an unstable partner/relationship; and jealousy from others’ desired fertility. Several reported that delaying pregnancy disclosure reduced their access to the financial and informational support they needed for earlier antenatal care initiation.

**Conclusions:**

Many pregnant Ugandan women preferred only disclosing their pregnancy to close, trusted social ties while delaying disclosure to others. Delays in disclosure affected the social support they received thereby contributing to late antenatal care initiation. Targeted interventions to address factors contributing to delayed pregnancy disclosure may improve the timeliness of antenatal care and consequent maternal and neonatal outcomes.

## Abstract (plain language summary)

Pregnant women in Uganda are not seeking health care as early as they should during pregnancy. This may be because they wait to tell family and friends who could help them get care for their pregnancy. Few studies have looked into when women share that they are pregnant with family and friends and how sharing this information could impact their access to care. We interviewed 30 pregnant women in Uganda and 15 male partners to explore their beliefs about when they should tell others about their pregnancy to help identify ways for women to access care earlier. We asked women who they told about their pregnancy and when, why they shared this information when they did, and if their decision to share this information was because they needed social support. Most women first only told close, trusted social family members like their partner and/or mother. Most preferred waiting until at least their second trimester to tell others about their pregnancy with many waiting to share this information for a long time. Reasons for waiting included fear of social judgement, witchcraft and curses, mockery and even anger which could cause adverse pregnancy outcomes. They were concerned about judgement of their age, lack of partner, or jealousy. A few respondents said they did not initiate antenatal care until they told their friends and family about their pregnancy. Delaying telling others reduced the help and information some respondents received to start antenatal care. Targeting reasons for delayed pregnancy disclosure could increase starting antenatal care early and impact maternal and infant health.

## Background

Maternal and neonatal mortality and morbidity are unacceptably high throughout many African countries [1, 2]. In Uganda, there are 189 maternal deaths per 100,000 live births and 52 infant deaths per 1,000 live births [3]. Early and appropriate use of antenatal care (ANC) is a crucial factor in reducing maternal and neonatal mortality and morbidity [4, 5]. ANC provides essential opportunities for prevention, detection and treatment of complications in pregnancy as well as opportunities for health education and support [6], including information about healthy behaviors in pregnancy, symptoms that warrant medical attention, malaria prophylaxis, deworming medication, iron supplements, insecticide-treated mosquito nets, tetanus shots, HIV testing and (if necessary) antiretroviral therapy, and social support [7].

Almost all pregnant women in Uganda (97%) have at least one ANC visit in pregnancy [8]. However, most pregnant women do not meet the World Health Organization’s 2016 recommendations of initiating ANC in the first trimester and having at least 8 contacts during pregnancy [5]. Only 29% of pregnant women in Uganda initiated ANC in the first trimester and median gestational age at ANC initiation is 4.7 months (~ 19 weeks) [8]. Findings from across Eastern and Southern Africa show that there are numerous barriers to early ANC initiation, including economic adversity, high parity, lack of social support (e.g. not receiving encouragement from family or friends to seek ANC or financial support), inaccessibility of health facilities, perceived poor quality of care and/or mistreatment by health care providers, cultural beliefs and knowledge gaps about the need for timely and regular ANC [9–14]. A study of 400 pregnant women initiating ANC late in pregnancy at Mulago Hospital found that the main contributing factors were lack of knowledge about ANC guidelines as well as insufficient financial resources for transportation and ANC service costs^1^ [10, 15].

Pregnant women may prefer to selectively disclose their pregnancy to certain social relations while preferring to delay or not disclose at all to others. They may also choose to phase their disclosure by sharing this information with certain social relations first, soon after they confirm they are pregnant, but delay disclosure to other social relations until later in the pregnancy, such as waiting until the second or third trimesters. Delays in disclosing pregnancy, meaning waiting to share that they are pregnant, can affect women’s access to social support (e.g. emotional, informational and financial support) which could create additional barriers to care-seeking in pregnancy. These potential delays in pregnancy disclosure are an under-investigated factor which could contribute to late ANC initiation in many African countries. A qualitative study in Malawi found that women preferred to hide their pregnancy in the first trimester due to fears that others finding out they were pregnant could lead to being bewitched causing harm to the mother and her baby [16]. Similarly in Uganda, delayed or non-disclosure of pregnancy is a common practice due to social and cultural beliefs that pregnancy disclosure could cause adverse maternal and neonatal effects through bewitchment by ill intended people in their communities [17]. In Zambia, a qualitative study among pregnant women and other key informants stated that disclosure of pregnancy is the duty of an older woman relative to other family members, and that attending ANC prior to the announcement will potentially expose the expectant mother to curses that could result in loss of the pregnancy [18]. A qualitative study also conducted in Uganda and South Africa identified delayed disclosure among HIV positive women in unstable relationships, with the disclosure delays depriving women of needed social support to seek timely ANC [19].

Delays in disclosing pregnancy can prevent pregnant women from accessing social support to facilitate early ANC initiation. Indeed, a growing body of evidence is demonstrating that pregnant women’s access to social support from their social networks (i.e., their partner, other family members, friends and others) is important for facilitating access to ANC services [10, 11, 20, 21]. We found that pregnant Ugandan women relied on female relatives (i.e., mothers, mothers-in-law, elders, sisters) and friends for information about when to initiate ANC [20], and that their social networks were instrumental in providing financial support so they could attend ANC [21]. Delays in pregnancy disclosure can also affect ANC initiation if pregnant women are not the sole decision-makers about their care-seeking. Late ANC initiators from the Mulago study reported that only 35% of them made the decision on their own of when to initiate ANC, while 41% made the decision together with their partner and 14% were instances where the partner made the decision alone [10]. Delays in disclosing pregnancy could also be due to delayed pregnancy confirmation, which has been shown to contribute to timing of ANC initiation [22]. Elucidating through in-depth, conceptualized data the beliefs and practices around pregnancy disclosure and their potential influence on timing of ANC initiation can reveal important intervention targets for increasing achievement of recommendations around ANC initiation.

To understand the role of pregnancy disclosure and timing of ANC initiation, we conducted a qualitative study among pregnant women and their male partners (for those in partnership) attending their first ANC visit at Kawempe National Referral Hospital, Uganda. We explored women’s decision-making around timing of pregnancy disclosure and ANC initiation, focusing on understanding reasons for delays in disclosing pregnancy, social norms related to pregnancy disclosure timing, and the role of pregnancy disclosure in timing of ANC initiation. Our findings can help inform culturally-appropriate interventions to effectively support women in initiating ANC in the first trimester, ultimately contributing to achieving Sustainable Development Goal 3 of ensuring health and promoting wellbeing at all ages, including reducing maternal, infant, and child mortality [23].

## Methods

### Overview of study design and recruitment

We conducted a qualitative study using semi-structured, in-depth interviews among 30 pregnant women and 15 male partners attending their first ANC visit at Kawempe National Referral Hospital (“Kawempe Hospital”). We purposively recruited the sample by partnership status meaning that half of the study participants identified as being in partnership. Among those study participants, we also recruited and interviewed their partner.

### Study setting

Kawempe Hospital is a national referral hospital serving metropolitan Kampala, Uganda and is the teaching hospital for Makerere University College of Health Sciences. It has 200 beds, with approximately 30,000 annual births with an outpatient ANC clinic serves approximately 200 clients per day [24, 25]. Services at Kawempe Hospital include surgical and non-surgical obstetrics and gynecology, newborn care, child and adolescent health, nutrition, and diagnostics (radiology and laboratory).

### Study participants

We recruited 30 pregnant women aged 18 years or older and attending their first ANC visit at Kawempe Hospital. We used purposive sampling to ensure that the perspectives of women in diverse partnership arrangements would be represented in the data. The use of purposive sampling meant that half of the pregnant women identified as being either married, engaged, or in non-marital partnership and half identified as having no current partner. We also interviewed male partners to understand their roles in ANC-seeking though their data are not included in this analysis. Due to restrictions on study recruitment during the COVID-19 pandemic, we were only allowed to recruit women who were attending their ANC visit with their partner because we could not ask partners to make a separate trip for interviews. The final sample size was determined based on thematic saturation from themes in the parent study related to social support.

### Participant recruitment, data collection and data analysis

Data collection and analysis were iterative. Interviews were conducted in Luganda, the local language in Kampala, between August and October 2020. Our research team worked together with the ANC nurses and midwives at the ANC clinic. Study participants were initially approached by the ANC nurses and midwives in the clinic waiting area prior to their ANC visit. If they expressed interest in participating in the study, the ANC nurses and midwives would then direct them to meet with our research team after their ANC visit to learn more about the study and complete the brief screening survey to determine eligibility and administer the consent process. Our study team conducted the interview in a private room after their ANC visit. All study participants provided written informed consent for us to conduct the interview and to obtain information from their medical records on gestational age at their first ANC visit. Following the consent process, we administered a short quantitative survey to collect data on socio-demographics, parity and other relevant data. We then conducted and recorded semi-structured, in-depth interviews which lasted 1–1.5 h. Coverage of specific interview content was facilitated with the use of interview guides that we created to ensure that study participants were asked about their decision-making regarding timing of ANC-seeking and the role of social support in facilitating access to care in pregnancy. We also included probes focused on women’s perspectives, experiences, and reasons for deciding when to disclose their pregnancy and how this affected timing of ANC-seeking. In the interview guide, we asked study participants to whom they disclosed their pregnancy once it was confirmed and whether they chose to delay disclosing their pregnancy from certain individuals in their social networks including the reasons for wanting to delay disclosure. We also asked how long they delayed disclosure once they confirmed their pregnancy and what reasons contributed to this. We probed for whether disclosure of the pregnancy to certain social ties was associated with wanting to access social support, including emotional, informational and financial support. We also asked about whether delayed disclosure to some social ties had implications for accessing social support contributed to timing of antenatal care initiation. We explored social norms around preferences to delay pregnancy disclosure and reasons behind these. The semi-structured nature of the interviews ensured that all participants commented on material specifically of interest to the study team while also permitting the interviewers to pursue new lines of inquiry or participants to address relevant issues that we may not have anticipated.

After the first 10 interviews were completed, the audio recordings were transcribed and translated into English. We reviewed these initial data and adapted the interview guides with additional probes and follow-up questions related to unexpected topics from the interviews. Once we completed all the interviews (*n* = 45), we transcribed the audio-recordings and translated them into English.

We used a thematic analysis with a two-stage coding process [26, 27], implemented in Atlas.ti 8.0 software. In the first stage, one of the coders read several transcripts to determine key concepts with potential codes and created a codebook with code definitions. The study’s primary analysis focused on different forms of social support to facilitate access to ANC [20, 21] and that analysis used both deductive and inductive codes related to the key domains for social networks and social support [28, 29]. In this current analysis, we focused on the sub-theme related to pregnancy disclosure. We planned to conduct analyses exploring women’s decisions to disclose their pregnancy to their social ties and the implications for accessing social support. Therefore, we had defined parent codes related pregnancy disclosure and the domains of social support following social network and social support theory. In reviewing the transcripts, we then added inductive codes based on sub-themes which emerged from the data related to timing of disclosure, delays in disclosure, and beliefs around reasons for delaying disclosure (such as fears and worries from disclosing early in the pregnancy).

To familiarize ourselves with the data and to identify these additional inductive codes from the data, three team members (AC, HN, TR) reviewed two transcripts and coded them independently. We met to harmonize the coding process and agree on the additional inductive codes. HN and TR independently coded 4 transcripts then met to ensure consistency in coding across transcripts, refine code definitions, and add additional codes. After code harmonization, HN and TR coded the remaining transcripts independently. The coding team met frequently to discuss and resolve any emerging issues in the data. Once coding was completed, HN led the analysis for this manuscript by identifying key concepts and themes, and the full team refined the analysis.

### Ethical approval

The study received ethical approval from Makerere University School of Medicine Research and Ethics Committee (reference number 2019–159) and the University of California San Francisco Institutional Review Board (reference number 19–27,163). We also obtained clearance to conduct the study from the Uganda National Council for Science and Technology (reference number SS5163). All participants provided written confirmation of informed consent. Study participants received 20,000 Ugandan Shillings (~ 5.25 US Dollars) for their participation.

## Results

Most women were nulliparous at the time of the interview (*n* = 19) and aged 25 years old or younger (*n* = 24). Owing to purposive sampling, half were in a stable partnership and half identified as having no current partner. At this first ANC visit, most study participants were in their second trimester of pregnancy (gestational age between 13 to 27 weeks). Only 6 were initiating ANC in the first trimester (gestational ages 12 weeks or less) and 6 were initiating ANC in their third trimester (gestational age 28 weeks or later) (Table 1). Among the sample of male partners, all were 20 years old or older (*n* = 15). About half had completed some secondary school (*n* = 8) with the remaining reaching higher than secondary school (*n* = 5). Only one had reached only primary school.

**Table 1:** Descriptive characteristics of study sample

	Women (*N* = 30)		Men (*N* = 15)	
	n	%	n	%
Demographics				
Age				
18–19	8	27	0	0
20–25	16	53	5	33
26–30	2	7	3	20
31 +	4	13	7	47
Parity				
0	19	63		
1	9	30		
2–4	0	0		
5 +	2	7		
Relationship status				
Married or living with partner	15	50	15	100
No partner	15	50	0	0
Highest schooling attained				
Primary	7	24	1	7
Secondary	17	56	8	57
Tertiary or more	6	20	5	36
Mean household size (SD)	4.1	(4.14)	3.9	(3.01)
ANC seeking				
Gestational age at first ANC visit *(from ANC card)*				
First trimester (< = 12 weeks)				
7 weeks	1	3		
10 weeks	1	3		
12 weeks	4	13		
Second trimester (13- 27 weeks)				
16 weeks	5	17		
17 weeks	1	3		
20 weeks	6	20		
24 weeks	1	3		
26 weeks	1	3		
Third trimester (28 + weeks)				
28 weeks	6	20		
30 weeks	4	13		

The in-depth interviews revealed that most pregnant women engaged in selective and phased disclosure of their pregnancy. The most common social relations that they first chose to disclose to were their partner (if partnered) and/or their mother, whereas they preferred to delay or avoid disclosing to other family members and friends including aunties, siblings, friends and neighbors. Pregnant women engaged in phased disclosure, by sharing this information with certain social ties (e.g. partner and mother) earlier than with others (e.g. siblings, friends and neighbors). Yet several mentioned even delaying disclosure to their partners and mothers due to concerns with their reactions. Most of the pregnant women in the sample wanted to delay disclosing their pregnancies at least until the second trimester with many preferring to wait for as long as they could. Some even preferred delaying disclosure until childbirth.

The study participants delayed disclosing their pregnancy primarily to mitigate judgement and related adverse consequences (being mocked or even scorned) and also to protect themselves from anticipated bewitchment or harm. Pregnant women in our sample cited several reasons why pregnancy disclosure could lead to these negative consequences: 1) they were seen by their community as too young to become pregnant and/or needing to prioritize school; 2) they were in a partnership that was not socially recognized and/or had poor partner relations; or 3) they faced jealousy from other individuals’ desired fertility. We found that pregnant women were reluctant to disclose their pregnancy both to people within and people outside their close social circles, even though this could have implications for ANC initiation. Indeed, our findings also showed that the tendency to delay pregnancy disclosure contributed to women not being able to access emotional, financial and informational support early in pregnancy which facilitates early ANC seeking.

We organized our findings under the following major themes: 1) disclosure of pregnancy within women’s social networks; 2) social norms around delaying disclosure of pregnancy; 3) negative consequences from pregnancy disclosure; 4) reasons behind the negative consequences from pregnancy disclosure; and 5) contribution of delayed pregnancy disclosure on late ANC initiation.

### Disclosure of pregnancy within women’s social networks

Most study participants engaged in selective disclosure of their pregnancy, by preferring to share this information only with certain social ties. Most commonly, pregnant women chose to first disclose their pregnancy to their partner (if partnered) and/or their mother.

One study participant explained:

“*Culturally, you shouldn’t tell many people that you are pregnant. You should only tell people close to you like mothers and husbands, but it is not good to tell people in the community about our pregnancies, unless it has reached a stage of being seen by people.”*

25-year-old female participant, partnered, 7 weeks pregnant at first ANC visit

Several confirmed that it was preferrable to avoid disclosure more broadly in their social networks and community. Certain social relations whom pregnant women wanted to avoid disclosing to included other family members and friends such as siblings, aunties, fathers, neighbors and some friends.

“*I wouldn’t tell my aunties…When I told them I was pregnant with my first baby their reaction wasn’t good. I think that maybe they didn’t like the man so imagine being pregnant for him again. Even some of my brothers might want me to be with someone else yet I chose this one. You understand? when they get to know that I am pregnant they will say, “She still got pregnant for him again!*”

24-year-old female participant, single, 28 weeks pregnant at first ANC visit

“*I can’t just come from no where and tell my friends that I am pregnant unless they get to find out on their own. I can’t tell them. They are not going to support me in anyway, they are just going to sit and start saying, “she did this and that*.”

19-year-old female participant, single, 12 weeks pregnant at first ANC visit

### Social norms around delaying disclosure of pregnancy

Most study participants delayed disclosing their pregnancy compared to when they knew they were pregnant. Many women and some male partners believed that pregnancy disclosure, if done at all, should happen selectively after the first trimester and should be limited to a few close and trusted people, such as their mothers and husbands/partners. They stated that other community members should learn about a pregnancy only when it becomes visible. Another study participant said that women should not disclose their pregnancy, further suggesting that it was culturally normative to delay disclosure. Instead, it was assumed that people would find out about the pregnancy mainly visually over time, “*No one should disclose that they are pregnant…It’s not hiding. You just don’t have to spread it everywhere*…” (22-year-old female participant, partnered, 28 weeks pregnant at first ANC).

Yet, several examples were also shared of delays in disclosure even to close social ties. Several study participants mentioned delaying disclosure to their mothers and relatives when they found out they were pregnant. Similarly, some also mentioned delays in disclosure to the partner.

*“I didn’t want my mother to know (that I’m pregnant) before our introduction ceremony. I was so afraid to tell her about it. I was 4 months (into pregnancy) when I told her. I was old enough and was working however, I was afraid of what my mother would think if I told her that I am pregnant for someone she doesn’t know.”*

24-year-old female participant, partnered, 20 weeks pregnant at first ANC visit

*“I told my friend (about my pregnancy way before) informing (the person who made me pregnant)…Because me and him were no longer in good terms. His brother was setting him up for another girl.”*

20-year-old female participant, single, 16 weeks pregnant at first ANC visit

### Negative consequences of pregnancy disclosure

Rather than describing how disclosure of pregnancy was accompanied by excitement and expectations of social support, study participants instead described how disclosing a pregnancy could lead to negative reactions from individuals in their social network, with concerns about mockery, judgement, and anger. In addition, there was significant concern about the possibility of being bewitched.

#### Concerns about mockery, judgement and anger

Both the female participants and the male partners interviewed explained that pregnant women were often called names or mocked by close social ties when they disclosed their pregnancies. One male participant explained what would happen if women disclosed their pregnancy:

*“People [make fun of pregnant women using] names like ‘mama tusuubira [expecting mother]’ and some men mock them with comments like, “Thank God for surviving the man [she had sex with]!” meaning it was some kind of rape or sex without consent. Those are some of the comments that they try to avoid [by not disclosing their pregnancies].”*

24-year-old male participant, partner of 20-year-old woman, 20 weeks pregnant at first ANC visit

Some study participants explained that, if they disclosed their pregnancies, people in the community would gossip about them and judge the putative timing. For example, short birth intervals (considered less than a year between birth and the next pregnancy) were not viewed as socially acceptable and could prompt judgment by others. One male participant elaborated on this phenomenon by saying: *The neighbors in our village judge and think that our first born is still very young for [us to have] a new pregnancy… It is such things [which make us hide pregnancy].”* (32-year-old male participant, partner of 22-year-old woman, 28 weeks pregnant at first ANC visit).

One female participant explained that she would be judged for the timing of her pregnancy, because her family and friends had advised that she delay pregnancy. She explained: “*My first reason [for not wanting to disclose the pregnancy] is that they [family members] had requested me not to get pregnant again and maybe wait for 2 years. However, we had agreed with my husband that we’ll try again.”* (27-year-old female participant, partnered, 10 weeks pregnant at first ANC visit).

Several of the study participants, primarily the young women under 20 years of age, shared their concerns about how others might be disappointed or even angered that they had become pregnant, to the extent that some wanted to hide their pregnancy until the delivery. One study participant shared that it would be more socially acceptable to appear with the new child rather than to appear as pregnant.

“*I didn’t want some people to know about my pregnancy. Some of them would get angry, I have studied the Qur'an so, I believe they would start mocking me saying, ‘How can she get pregnant yet she pretends to be religious?’ so I realized that it’s better to [hide the pregnancy] and go with a [born] child home than with a pregnancy.*”

18-year-old female participant, single, 24 weeks pregnant at first ANC visit

Women were not only worried about anticipated anger and scorn from community members, but they were also concerned about similar reactions from close social ties, such as their own mothers. One study participant said she delayed disclosing her pregnancy to her mother for as long as possible:

*“It took some time for my mother to know that I was pregnant. I never wanted to tell her because I knew she will be mad about it, she would say, ‘How can you get pregnant when you can't even take care of the first born?’ She used to ask me if I was pregnant and I would deny it until it was seen.***”**

18-year-old female participant, single, 30 weeks pregnant at first ANC visit

Another female participant, who had rheumatic heart disease, was concerned that her mother would react negatively because of the potential maternal and fetal risks. She shared: *“I took a long time to tell my mother because I thought that she will quarrel, but she didn’t.”* (23-year-old female participant, partnered, 12 weeks pregnant at first ANC visit).

#### Concerns about bewitchment

Several female participants and their partners explained that they wanted to delay or even avoid disclosing their pregnancies because they were worried about the ill intentions of others. They believed certain people were capable of bewitching or cursing them, or of deliberately providing harmful advice that could result in adverse maternal outcomes such as miscarriages or even future infertility. These people were often identified as being neighbors living close proximity or even family members. One female participant explained her concerns:

*“It is not good to talk to people about how old the pregnancy is. Among the people you tell, [there] are those who don’t wish you well and those who can do something wrong. We hear a lot of people saying, ‘Someone tied my pregnancy [carrying a pregnancy beyond term]’ or ‘Someone was bewitched to be barren.’ Those are the reasons as to why people don’t like to make their pregnancies public to just anyone.*”

25-year-old female participant, partnered, 7 weeks pregnant at first ANC visit

Another female participant shared similar concerns about what neighbors could do: “*Neighbors have evil hearts and don’t wish anything good for you…They can do something bad to your pregnancy…giving you things with herbs in them to cause an abortion*.” (20-year-old female participant, partnered, 20 weeks pregnant at first ANC visit).

One male partner explained:

*“Some of them (pregnant women) come from families that practice witchcraft, so they hide their pregnancies in fear that they might be bewitched…so, they will try to hide the pregnancy and maybe reveal it when it is at 3 months.”*

33-year-old male participant, partner of 23-year-old woman, 16 weeks pregnant at first ANC visit

### Reasons behind negative consequences from pregnancy disclosure

Study participants explained that there were several reasons why they would face these negative consequences from disclosing their pregnancies. These reasons included: being considered too young to become pregnant or leave school, being in a partnership that was not socially approved, having a poor relationship with their partner, and facing jealousy from others’ fertility desires.

#### Social norms about being considered too young to become pregnant or leaving school

If the woman was considered too young to be pregnant based on social norms and expectations, or if she was still in school, she would often prefer delaying pregnancy disclosure to avoid adverse reactions. One female participant said *“I was afraid [to disclose my pregnancy]. They [family and others] would say that I got pregnant while young.”* (18-year-old female participant, single, 30 weeks pregnant at first ANC visit).

Many study participants, especially the women, shared that they would wait until the pregnancy was visible before disclosing it, as they worried about being reprimanded. One female participant explained that Ugandan families placed high expectations on young women who were still in school:

*“It also depends on age; I was a finalist [in the final year of school] so people would say, ‘She hasn’t finished school and now she is pregnant.’ I wasn’t living with mother but with my uncle and [his] wife. It was my uncle paying my school fees, so we hadn’t told him about it because we were afraid what he would say. Everyone thought that I had done a bad thing because I hadn’t yet done my finals examinations…You know how people think that children who are at the university are going to transform their families; I was scared that she would feel disappointed in me.”*

22-year-old female participant, partnered, 20 weeks pregnant at first ANC visit

Another study participant who was concerned about anger and physical abuse from her parents if she had disclosed her pregnancy earlier on said: *“[My parents] could have beaten me [if I told them earlier about my pregnancy]. I was doing a practical course in school*.” (20-year-old female participant, partnered, 12 weeks pregnant at first ANC visit).

#### Social norms related to partnership and quality of relationship

Unlike the male participants in the study, many of the women – both partnered and not partnered – felt that a pregnancy would be accepted if the woman was in a socially sanctioned relationship, such as a legal marriage or stable partnership following social norms. If a pregnant woman was not in such a relationship, she would likely face reprimand and ostracism and might therefore prefer not to disclose her pregnancy. Women who were no longer partnered at the time of the interview shared that pregnancies were often not disclosed if they were unplanned or happened at a time when partner relations were worsening. One female participant said: *“I never wanted anyone in my family to know that I am pregnant because they would ask for my husband, yet we are not together anymore.”* (22-year-old female participant, single, 20 weeks pregnant at first ANC visit).

As another study participant explained, a pregnant woman would delay disclosing her pregnancy for as long as possible if the partner was not acknowledging the pregnancy.

*“You can get pregnant and the man responsible denies it so, you have to hide it until you are about to give birth so that [your family members] don’t send you away from home [from having become pregnant when not married].”*

23-year-old female participant, partnered, 16 weeks pregnant at first ANC visit

#### Jealousy leading to bewitchment and miscarriage

Most of the study participants, especially the male partners in the study, also mentioned that pregnant women preferred to delay pregnancy disclosure because they feared being bewitched by people who might be jealous of them. This jealousy was often attributed to others wanting to become pregnant and facing challenges with infertility and miscarriages. Disclosing a pregnancy could be viewed by others as bragging or showing off. One male participant said: “*When you share that you are pregnant, one can go and do something evil to you. They will harm you because they feel like you are bragging for being pregnant.”* (32-year-old male participant, partner to 22-year-old female participant, 20 weeks pregnant at first ANC visit).

Another male participant elaborated on this notion of jealousy and unmet fertility within the community:

*“There are people that are so evil that they can comment things like, ‘Hmm! She thinks she’s the first person to have a child?’ So a woman may be forced to hide her pregnancy because of such people... One can be like, ‘Who is she to have a child and yet girls in my family have not been able to have children for years?’ In fact, an evil person can curse your pregnancy and then you find yourself being pregnant for a full year! With just evil words. Other people just have a ‘bad eye’ while others are ‘witches’… that is why people say that it is wise not to show everyone your pregnancy.”*

31-year-old male participant, partner to 24-year-old female participant, 28 weeks pregnant at first ANC visit

Bewitchment was often attributed to this jealousy from within the community and would explain adverse outcomes in pregnancy such as miscarriages. One female participant shared:

“*People don’t wish you well and are jealous. Because of this, the community believes it’s not good to let people know that you are pregnant…during the first trimester it is easier to have a miscarriage because anyone can do something and you lose the pregnancy. You could jump [meaning get in contact unknowingly with] herbs that harm the baby. The risks reduce during the 2nd and 3rd trimester.”*

27-year-old female participant, single, 10 weeks pregnant at first ANC visit.

#### Contributions of delayed pregnancy disclosure to late ANC initiation

The delays in pregnancy disclosure appeared to affect when pregnant women initiated ANC, especially for women who were young or pregnant for the first time and who had no prior experience with ANC. For example, when asked what would have helped her seek ANC earlier, one 18-year-old woman who had an unintended pregnancy while in school, said that she would have wanted to disclose her pregnancy earlier. She was 6 months pregnant at her first ANC visit. She explained: *“I wish I talked [earlier to my mother] instead of being quiet…I feared my mother would have chased me from home.”*(18-year-old female participant, single, 30 weeks pregnant at first ANC visit).

Another study participant who was also pregnant for the first time attributed her late initiation of ANC, at 20 weeks pregnant, to her delay in disclosing her pregnancy to her family. She explained her delay by describing that she had limited knowledge about the need for ANC.

*“I started late on my antenatal care because I didn’t know anything about it and… my sister got to know about it [my pregnancy] late… When I told my sister about it [my pregnancy], she gave me money to come here. I didn’t know that I was supposed to be taking tablets after doing the [ultrasound] scan.”*

23-year-old female participant, single, 20 weeks pregnant at first ANC visit

Despite all of these reasons to delay disclosure of pregnancy, many study participants viewed disclosing their pregnancy as a way to obtain social support from their partner, their family, and others. As emphasized by one study participant: “*It can’t cross my mind [to keep quiet about the pregnancy] because I so much need his [partner’s] support. I can’t hide it*.” (23-year-old female participant, partnered, 12 weeks pregnant at first ANC visit).

Indeed, one woman who was financially dependent on her partner explained how delaying disclosure of her pregnancy to her husband would leave her with insufficient financial support to access care; however, she felt conflicted about telling her partner because she had become pregnant before her partner was ready for a child.

*“I didn’t want to disclose to my husband because I conceived before we agreed (to have a baby). So that made it hard for me because I didn’t have money [to go for ANC] and yet he is the one I must ask for it.”*

19-year-old female participant, partnered, 26 weeks pregnant at first ANC visit

The importance of financial assistance was emphasized by another woman who felt she needed to disclose her pregnancy to her parents so that she would have the means to access care. She shared: *“When I got pregnant, I told myself that I can't hide it from my parents because they can help me when no one else can.” (*18-year-old female participant, single, 28 weeks pregnant at first ANC visit).

This study participant’s delayed initiation of ANC despite her acknowledgment of the benefits of disclosing her pregnancy to her parents suggests that other social norms also contribute to late ANC initiation.

## Discussion

In this qualitative study of pregnant women and their partners in Uganda, we explored their perspectives around who they disclosed their pregnancy to and who they wanted to avoid disclosing to. We also investigated the timing of pregnancy disclosure relative to when they knew they were pregnant and the implications, if any, for initiation of ANC. Our findings revealed that pregnant women selectively disclosed their pregnancy, mostly first to their partner and/or their mother while avoiding disclosure to other social relations such as neighbors, other family members and friends. The findings also showed that pregnant women and their partners preferred to wait until at least the second trimester before disclosing their pregnancies and they often did so only to close, trusted social relations. Women delayed disclosure because they were concerned about being subjected to mockery, judgment, anger, or bewitchment, the latter of which was associated with concerns about potential physical harms (i.e., for the mother/pregnancy or for the fetus). These anticipated, negative consequences were linked to social judgement due to social norms around when and in what type of relationship a woman was expected to become pregnant (e.g., seen as too young, still in school, or not in socially recognized relationships) and jealousy from others’ desired fertility. We found that these delays in pregnancy disclosure contributed to late ANC initiation, partly because women failed to access social support from their close social relations to facilitate ANC-seeking. Given the importance of timely ANC to both maternal and child outcomes, our findings about delays in pregnancy disclosure have important implications for public health.

Desires to delay disclosure of pregnancy were common among study participants. This finding is consistent with the findings from other studies, both in Uganda and in other African countries. In Uganda, pregnant women tend to prefer delaying disclosure of pregnancy for as long as possible, or not disclosing the pregnancy at all, due to concerns about being subjected to witchcraft, potentially resulting in adverse maternal outcomes [17]. In Uganda, there exists widespread belief in the existence of evil spirits which are seen as responsible for bringing misfortune, including causing ill health and death [30]. Attributing adverse pregnancy outcomes to witchcraft and evil spirits – and the concomitant desire to delay disclosure – has indeed been found in several other African countries, including in Malawi [16], Tanzania [31], Kenya [32], and the Gambia [33]. In two studies, one from Tanzania and the other from Kenya, concerns about vulnerability to witchcraft and spirits contributed to pregnant women’s preferences for seeking care through traditional healers and thereby delaying ANC-seeking in formal settings [32, 34].

Another reason for delaying pregnancy disclosure related to concerns about adverse social consequences (e.g., being called names and gossiped about). Study participants expressed worry that others would judge them for being too young to be pregnant, for getting pregnant while still in school, or for getting pregnant despite not being in socially sanctioned relationships consistent with social norms in this context. The quality of the relationship also contributed to whether women preferred delaying disclosure, especially in cases where the male partner denied the pregnancy.

The existence of these cultural and social norms around who is expected to get pregnant with whom or when is a socially acceptable time to become pregnant leads women to delay disclosure of their pregnancy and prevents them from having a trusted confidant to rely on for support, especially in the early stages of the pregnancy. As a result of delaying disclosure, pregnant women also delay any support they may receive from close social ties. There were several examples of women delaying ANC because they lacked financial assistance. Indeed, only half of married women in Uganda make their own financial decisions [8] and financial dependency on others within the household, including their partner and/or parents, infringes on their autonomy in care-seeking. Other research found that over half of the pregnant women who initiated ANC late in pregnancy at Mulago Hospital in Kampala, Uganda were instances where either the partner was solely responsible for care-seeking decisions or the couple jointly decided [10]. Our results are consistent with other studies examining factors contributing to delayed ANC initiation, including one study finding that delayed disclosure was often due to concerns over social stigma and shame from becoming pregnant outside of marriage [33]. Other studies confirm that important role of social networks in providing financial support to facilitate timely initiation of ANC [13, 21].

Our findings suggest several next steps for supporting pregnant women to access ANC earlier in pregnancy, despite social norms of delaying disclosure. Sensitization campaigns that ensure pregnant women, as well as their social networks, know the recommended timing of ANC and the benefits of early ANC initiation and can identify the signs of complications in pregnancy are critical. Efforts should also be made towards reducing stigma aimed at women who become pregnant outside of socially accepted circumstances (e.g. while still in school, at a young age, not partnered) and disseminating information about the adverse effects of this stigma such as delays to ANC initiation because they lack the necessary social support. At the same time, there is a critical need to ensure access to reproductive health services so that women can exercise their reproductive autonomy in deciding if and when to become pregnant. Village health teams and community health workers could be engaged to identify pregnant women early in pregnancy to encourage ANC-seeking, though this is challenging given preferences to delay disclosure of pregnancy. Alternatively, village health teams and community health workers could engage women’s social networks (e.g. mothers, sisters, female friends, elder women) to spread information on the importance of early ANC-seeking. Improving economic opportunities for women could also be a promising way to ensure that they have greater decision-making power related to care-seeking, including during pregnancy.

### Limitations

The findings of our qualitative study are not generalizable to all pregnant women in Uganda, yet they provide a nuanced understanding of the social and cultural norms around delays in pregnancy disclosure, reasons for these delays and the implications for ANC initiation. Interpretation of our findings must take into consideration the sociodemographic characteristics of our study participants. The study was conducted in Kampala, the capital city of Uganda; in this major urban area, access to care is better and the socio-economic status of study participants may be higher compared to women and men from more rural areas. A second limitation is that the study was conducted among women who decided to seek ANC services. Their views and experiences may be different from pregnant women who have more limited access to care and women who present for ANC at even later stages in pregnancy. The required conditions of recruitment during the COVID-19 pandemic were such that our sample of pregnant women in partnership was restricted to those who attended ANC services accompanied by their male partner. Women who were accompanied to the visit by their partner may receive greater partner support and this may further suggest that their pregnancies were less like to be unintended or mistimed, both of which could influence their perspectives about delaying disclosure and their antenatal care seeking behaviors. While data from these study participants suggested that about half experienced some initial ambivalence about their pregnancy and considered terminating, the potential interaction between partner support and pregnancy intentions on disclosure should be explored in future quantitative research. The women participating in our study also tended to be nulliparous (since anecdotally Uganda women in stable partnership tend to attend ANC alone for subsequent pregnancies). Furthermore, our study reflects women ages 18 years or older, yet it would be important to understand whether delays in disclosing pregnancy are even more prevalent among younger women and the implications for accessing social support and initiating ANC early.

## Conclusions

We found that pregnant women in Uganda preferred disclosing their pregnancy only to close social relations such as their partner and mother. Yet even with these close social ties, there were often delays in pregnancy disclosure. Although pregnant women could benefit from activating their social networks early in pregnancy to access financial, informational and emotional support, the decision to delay pregnancy disclosure adversely affected their ability to seek early ANC. Drawing attention to reasons for delaying pregnancy disclosure even to close social relations is critical to ensure women access ANC earlier in pregnancy and ultimately improve maternal and neonatal health outcomes. Targeted efforts are needed to improve reproductive autonomy (e.g. through access to contraception and economic empowerment more generally) and to mitigate the negative social consequences faced by women who become pregnant in non-socially sanctioned contexts. Public health campaigns are also needed to improve knowledge about the recommended timing of ANC, the benefits of early ANC initiation, and the signs of pregnancy complications. Structural interventions are also important to engage community-level actors in the existing national health system structure, the village health teams, other community members, and traditional birth attendants to support early ANC initiation, especially in contexts where beliefs in witchcraft can lead pregnant women to disengage from their social networks during early stages of pregnancy. Taken together, our findings suggest that interventions that foster greater social support for pregnant women to support earlier care-seeking may make a significant difference in achieving better health for women and their children.

## Data Availability

The datasets used and/or analyzed during the current study are available from the corresponding author on reasonable request.

## Abbreviations

ANC: Antenatal care
HIV: Human immunodeficiency virus

## Footnotes

1 Despite Uganda’s formal maternal health care user exemption policy, typical patient costs during the ANC visit can include: fee for ultra-sound (the average user fee for ultrasound services at a public facility. was (13,928 UGX ~ 3.66 US dollars) was less than that at private non-profit facilities (23,402 UGX ~ 6.16 US dollars) and at private for-profit facilities 39,486 UGX ~ 10.39 US dollars), supplies (e.g. gloves), and dressing gown.
