# Correction: Delayed pregnancy disclosure, attributed social factors and implications for antenatal care initiation: a qualitative study among Ugandan women and their partners

**DOI:** 10.1186/s12978-025-02062-6

**Published:** 2025-06-25

**Authors:** Hadija Nalubwama, Alison M. El Ayadi, Cynthia C. Harper, Josaphat Byamugisha, Dilys Walker, Alexander C. Tsai, Blake Erhardt-Ohren, Umar Senoga, Paul J. Krezanoski, Carol S. Camlin, Alison B. Comfort

**Affiliations:** 1 Department of Obstetrics and Gynaecology, College of Health Sciences, School of Medicine, Makerere University, PO Box 7072, Kampala, Uganda; 2 Department of Obstetrics, Gynecology and Reproductive Sciences, Bixby Center for Global Reproductive Health, University of California San Francisco, 550 16 th Street, San Francisco, CA 94143 USA; 3 Center for Global Health and Mongan Institute, Massachusetts General Hospital, Harvard Medical School, 125 Nashua Street, Suite 722, Boston, MA 02114 USA; 4 University of California Berkeley, 2121 Berkeley Way, Berkeley, Berkeley, USA; 5 University of California San Francisco, Zuckerberg San Francisco General Hospital, 1001 Potrero Ave, San Francisco, CA 94110 USA; 6 Department of Obstetrics, Gynecology and Reproductive Sciences, Bixby Center for Global Reproductive Health, University of California San Francisco, 1330 Broadway, Suite 1100, Oakland, CA USA

**Correction** **:** **Reproductive Health 22, 90 (2025)**

**https://doi.org/10.1186/s12978-025-02040-y**

In this article, the statement ‘the National Institute on Drug Abuse (K24DA061696)’ was missing in the Funding information section and has been included.

In addition, the statement ‘ACT reports receiving a financial honorarium from Elsevier for his work as Co-Editor in Chief of the Elsevier-owned journal SSM-Mental Health’ was missing in the competing interests section and has been included.

The complete funding and competing interests sections should read as below.

